# “It's That Route That Makes Us Sick”: Exploring Lay Beliefs About Causes of Post-traumatic Stress Disorder Among Sub-saharan African Asylum Seekers in Germany

**DOI:** 10.3389/fpsyt.2018.00628

**Published:** 2018-11-26

**Authors:** Freyja Grupp, Marie Rose Moro, Urs M. Nater, Sara M. Skandrani, Ricarda Mewes

**Affiliations:** ^1^Division of Clinical Biopsychology, Department of Psychology, University of Marburg, Hesse, Germany; ^2^University of Paris Descartes, Hospital Cochin Paris, Paris, France; ^3^Division of Clinical Psychology, Faculty of Psychology, University of Vienna, Vienna, Austria; ^4^University of Paris Nanterre, Hospital Cochin Paris, Paris, France; ^5^Outpatient Unit for Research, Teaching and Practice, Faculty of Psychology, University of Vienna, Vienna, Austria

**Keywords:** asylum seekers, causal beliefs, post-traumatic stress disorder, refugees, Sub-Saharan Africa, trauma

## Abstract

Many asylum seekers have been confronted with traumatizing events, leading to high prevalence rates of post-traumatic stress disorder (PTSD). Within the diagnostic context, clinicians should take into account patients' culturally shaped presentation of symptoms. Therefore, we sought to provide insights into beliefs about causes of PTSD held by Sub-Saharan African asylum seekers living in Germany. To this aim, we used a quantitative and qualitative methodological triangulation strategy based on a vignette describing symptoms of PTSD. In the first part of the study, asylum seekers (*n* = 119), predominantly from Eritrea (*n* = 41), Somalia (*n* = 36), and Cameroon (*n* = 25), and a German comparison sample without a migration background (*n* = 120) completed the Revised Illness Perception Questionnaire (IPQ-R). In the second part, asylum seekers reviewed the results within eight focus group discussions (*n* = 26), sampled from groups of the three main countries of origin. Descriptive analyses of the first part demonstrated that asylum seekers predominantly attributed PTSD symptoms to psychological and religious causes, and rather disagreed with supernatural causes. In comparison to the German sample without a migration background, asylum seekers attributed symptoms less strongly to terrible experiences, but more strongly to religious and supernatural causes. Within the focus group discussions, six attribution categories of participants' causal beliefs were identified: (a) traumatic life experiences, (b) psychological causes, (c) social causes, (d) post-migration stressors, (e) religious causes, and (f) supernatural causes. Our findings suggest that the current Western understanding of PTSD is as relevant to migrants as to non-migrants in terms of psychological causation, but might differ regarding the religious and supernatural realm. While awareness of culture-specific belief systems of asylum seekers from Sub-Saharan Africa regarding PTSD is important, our findings do underline, at the same time, that cultural differences should not be overstated.

## Introduction

Since 2011, global displacement and migratory movements within the African continent have grown annually (1). Within the last 5 years, the total number of displaced people in Africa doubled to approximately 20 million (1). Multiple crises across the continent remain unresolved and displacement and massive resettlement is highly likely to continue (1, 2). Refugees and asylum seekers from Sub-Saharan Africa arriving in Western countries constitute a particularly vulnerable group, who might have been confronted with an exceptionally high number of traumatizing events, such as torture, sexual violence, war, and armed conflict (3–5). These traumatic experiences often lead to high prevalence rates of post-traumatic stress disorder (PTSD) (6–11).

According to the 10th International Classification of Diseases [ICD-10; (12)] and the 5th Diagnostic and Statistical Manual of Mental Disorders (DSM-5), (13) PTSD is characterized by a constellation of symptoms thought to result from exposure to one or more traumatic events. Typical features include episodes of repeated reliving of the trauma in intrusive memories (“flashbacks”), dreams or nightmares, occurring against the persisting background of a sense of “numbness” and emotional blunting. Furthermore, detachment from other people, unresponsiveness to surroundings, anhedonia, and avoidance of activities and situations reminiscent of the trauma arise. Usually, persons with PTSD also suffer from a state of autonomic hyperarousal with hypervigilance, an enhanced startle reaction, and insomnia (12).

Several theories have been presented to explain the development of PTSD and cognitive models have offered a useful conceptualization. Janoff-Bulman's (14) theory of shattered assumptions assumes a worldview consisting of underlying assumptions about beliefs in the self and the world, as a just, benevolent, and predictable place in which the individual possesses competence and worth (15). According to the theory, these assumptions are undermined, or shattered, by the experience of trauma. As a result, individuals no longer perceive the world as benevolent and predictable or themselves as competent and invulnerable. The subsequent state of defenseless and frightening awareness of personal vulnerability gives rise to the symptoms characterizing PTSD (14, 15). According to Janoff Bulman's (16) traumatic model of shattered assumptions, causal beliefs are involved in reconstructing the shattered assumptions in the aftermath of extreme experiences. Causal beliefs about mental illness involve attribution processes that play a significant role in granting people a sense of prediction and control over their lives (16–18). As they are forming cognitions and guiding behavior, causal beliefs play an essential role in shaping illness experience in different sociocultural groups (19–23). The cross-cultural understanding of illness beliefs is based on Kleinman's (24) research in the fields of anthropology and medicine. Past research has emphasized a seemingly dichotomous view regarding causal beliefs of mental disorders, dividing cultures into belonging to the Global South or the Global North (25). Accordingly, perceptions of mental disorders in cultures of the Global North are predominantly shaped by multi-causal beliefs combining biological, genetic, and psychosocial explanations with environmental factors and stressful life events (26–28). In cultures of the Global South, religious, magical, and supernatural causal beliefs can be encountered as well (22, 29, 30).

Despite their cultural and religious diversity, different cultures of Sub-Saharan Africa show remarkable similarities regarding their causal attributions of mental distress. The literature has described misuse of psychoactive substances and biomedical and psychosocial explanations as causes of mental disorders, (18, 31, 32) but has particularly emphasized the role of religion and supernatural phenomena (31, 32). Quantitative research in samples from West Africa, (27) Cameroon, (33) and Nigeria (26) found that compared to Western samples, African respondents were more likely to attribute mental disorders to supernatural causes. In Sub-Saharan African cultures, a general belief in external causation by supernatural phenomena such as sorcery, spiritual possession, and being cursed or bewitched seemed to be highly relevant in explaining mental suffering (18, 25, 34). With regard to refugees and asylum seekers from Sub-Saharan African backgrounds in Western resettlement countries, traditional causal beliefs about mental illness may vary due to immigration and acculturation processes (35, 36). Additionally, post-migration stressors, social isolation, and living in exile may emerge as new causes of mental distress (35, 37–39).

While past research on causal beliefs in Africans focused mainly on symptoms of schizophrenia, depression, or mental disorders in general, (26, 27, 34) little is yet known about causal beliefs regarding PTSD.

As Western health care professionals are increasingly confronted with patients from diverse cultural backgrounds, clinicians need to consider patients' culturally shaped belief systems. It cannot be supposed that patients share the Western cultural concepts and values (40). Therefore, elucidating causal beliefs about mental disorders might improve the diagnosis of patients from different cultural backgrounds and minimize cultural distance. Such insights should in turn encourage Western practitioners to employ culturally sensitive treatment strategies instead of imposing Western concepts, which might not be suitable for the patients' belief systems (31, 41).

Moreover, causal beliefs influence treatment-seeking and health care utilization (35, 38). Disparities in mental health care for asylum seekers and refugees have been observed (38, 42), and have been attributed to a large extent to clinicians' deficits in knowledge and lack of cultural competence (35, 43, 44). Enhancing knowledge of causal beliefs might therefore be one step toward better mental health care provision for refugees and asylum seekers.

Against this background, the present paper aims to examine lay causal beliefs of asylum seekers from Sub-Saharan Africa in Germany regarding the symptoms of PTSD, and whether supernatural and religious causations play a relevant role in explaining PTSD in this group. Furthermore, our study focuses on the comparison of causal beliefs of PTSD held by Sub-Saharan African asylum seekers and those held by a German population without a migration background. We argue that an analysis based on the examination of similarities and differences can be one step forward to tackle an ethnocentric practice in mental health care (45).

We examine the following hypotheses: There is a difference between Sub-Saharan African asylum seekers and participants without a migration background concerning causal beliefs of PTSD symptoms. While asylum seekers attribute PTSD more strongly to religious and supernatural causes, participants without a migration background indicate a stronger belief in psychological causes. To account for possible influences of countries of origin on causal beliefs, group differences between asylum seekers from main countries of origin were explored.

The study aims were addressed using a qualitative and quantitative methodological triangulation strategy. Quantitative designs in cross-cultural research may face the problem of transferability of theoretical constructs, as Western theories and concepts might be ill-suited to explain the understanding and reasoning of individuals from non-Western cultural backgrounds (46). Therefore, the exclusive use of quantitative methods to generate meaningful data is problematic. Qualitative designs, however, are suitable for generating in-depth and detailed descriptions of constructs, and address subjective meanings, but they often remain “stuck” at the descriptive level (46). We therefore argue that a combination of these two research methods is appropriate to address our research aims, because it enables us to decrease the weaknesses of one individual method and strengthen the outcome of the study (47). In this respect, the findings from each method are complemented by the other (47) and a culturally sensitive interpretation of the results is ensured.

## Methods

### Procedure

The study consisted of two parts: (a) a questionnaire survey in which participants responded to the Revised Illness Perception Questionnaire (IPQ-R) causal scale (48) and (b) focus group discussions in which respondents reviewed the results of the questionnaire survey. Ethical approval was obtained from the local review board for each part of the study and all participants provided informed consent prior to participation.

Inclusion criteria for study participation of asylum seekers were refugee or flight experience, an origin in a Sub-Saharan African country, and age over 18 years. Participants in the group without a migration background had to have been born in Germany and have no be without any migration background. Individuals who had studied medical and psychological subjects and those who had worked in a health profession were excluded.

In the first part of the study, a convenience sample of *n* = 120 German participants without a migration background, as well as *n* = 119 asylum seekers from seven Sub-Saharan African countries, mainly Eritrea (*n* = 41), Somalia (*n* = 36), and Cameroon (*n* = 25), took part in a questionnaire survey. The group of asylum seekers was approached by applying a combination of convenience and snowball sampling. They were recruited in their accommodation facilities, through collaboration with civic refugee initiatives, from language courses for adult immigrants, and by networking at religious and cultural gatherings. Data were collected between April and December 2016 throughout different cities in Germany using paper-and-pencil and online assessments (survey software UNIPARK & QuestBack). The questionnaire survey was made available in German, English, French, Tigrinya, and Arabic.

In the second part of the study, 26 participants reviewed the findings of the questionnaire survey in focus group discussions. Given that the first sample consisted mainly of individuals from Eritrea, Somalia, and Cameroon, members of the focus group discussions were sampled according to these three countries of origin. Eight focus groups were conducted with participants from Eritrea (*n* = 10; three focus groups), Somalia (*n* = 8; three focus groups), and Cameroon (*n* = 8; two focus groups). The discussions took part in prepared interview rooms throughout different cities in Germany. The average duration of the focus group discussions was 1 h 30 min. The first author, a trained clinical psychologist, conducted the focus group discussions in English and French. For two focus groups with participants from Eritrea, she was supported by bilingual Tigrinya-German interpreters, who received detailed instructions beforehand. During the focus groups, the moderator invited participants to share their opinions actively and assured that each participant had an equal chance of expressing his/her views. The discussions were audio-taped and supplemented by hand-written notes.

### Measures and materials

Participants of both groups were asked to provide demographic information in each part of the study (see data in Table 1).

**Table 1:** Study sample and characteristics.

**Variable**	**Asylum seekers** ***n*** = **119**		**Participants without migration background** ***n*** = **120**				**Sample of the qualitative part** ***n*** = **26**		
	**M (SD)**	**range**	**M (SD)**	**range**	***t***	***p***	**Eritrea (*n* = 10)**	**Somalia (*n* = 8)**	**Cameroon (*n* = 8)**
Age (years)	27.97 (7.8)	18–54	37.24 (16.5)	18–79	−5.56	< 0.001	31.9	25.6	23.4
Education (years)	9.7 (3.6)	2–18	12.2 (1.7)	6–16	−6.64	< 0.001	10.8	5.6	9.5
					χ2	***p***
Males	70.7%		35.0 %		30.1	< 0.001	80%	87.5%	100%
Education					36.51	< 0.001		
university degree	13.4 %		33.3 %	
higher–education entrance–level qualification	18.5 %		30 %		
secondary school certificate	39.5 %		31.7 %	
primary school education	21.0 %		1.7%		
no school-leaving qualification	5.0 %		1.7 %		
Religion					63.7	< 0.001
Christianity	56.3 %		65.8 %
Islam	33.6 %		0.8 %	
Judaism	1.7 %		0.8 %	
No Religion	4.2 %		31.7 %
Other	0.8 %		0.8 %	
					***t***	***p***
Importance of faith	2.90 (0.50)	0–3	1.52 (1.32)	0–3	10.65	<0.001
Assistance from faith	3.67 (0.94)	0–4	1.84 (1.60)	0–4	10.74	<0.001
**Country of birth**									
Eritrea	*n* = 41 (34.5 %)
Somalia	*n* = 36 (30.3 %)
Ethiopia	*n* = 7 (5.9 %)	
Sudan	*n* = 2 (1.7 %)	
Cameroon	*n* = 25 (21.0 %)
Nigeria	*n* = 4 (3.4 %)
Togo	*n* = 4 (3.4 %)
Posttraumatic symptom severity score	15.25 (10.42)	0–41	7.09 (9.76)	0–36	5.88	<0.001
					χ2	**p**
Number of experienced traumatic events	5.02 (4.91)	0–19	1.33 (2.03)	0–11	62.34	< 0.001
Prevalence of at least one traumatic event	73.9 %		54.2%

In the questionnaire survey, participants were presented with a standardized unlabeled vignette that was integrated in the questionnaire. During focus group discussions in the second part of the study, the same vignette was read aloud and additionally disseminated in printed form. The vignette illustrated a hypothetical friend describing symptoms of PTSD according to the criteria outlined in the ICD-10 (12, 49). Following criterion A of ICD-10 (12), a stressful event of exceptionally threatening nature was presented as triggering event for symptoms. It was adapted for asylum seekers and participants without a migration background in order to improve the fit to their living situations and the possibility of experiencing such a traumatic event. Whereas “flight” was a typical traumatic event experienced by asylum seekers, physical violence was among the most commonly experienced traumatic events in the German general population (50). Therefore, we used flight as event in the vignette for asylum seekers and operationalized physical violence with the event “robbery” in the vignette for Germans without a migration background. In accordance with typical symptoms of PTSD described in criteria B to D in ICD-10, the vignette included the occurrence of insomnia, flashbacks, and nightmares, as well as a state of hypervigilance, senses of numbness and detachment from other people, anhedonia, and unresponsiveness to surroundings (12).

The vignette read as follows:

“*Since the [flight/armed robbery] I became a totally different person. In the evenings I lie in bed and then come these thoughts and images and I lie awake forever. Now I have reached a point where I notice I can't go like this anymore… Sometimes I scream at night and I wake up drenched in sweat because of the nightmares. If I have arrived somewhere and there is a noise, I wince. There it is again. I can't turn it off, it's like an electric shock that immediately goes straight up and triggers intense sweating. My wife/My husband accuses me of often being aggressive, easily irritable and she/he is afraid of my outbursts of rage. That's why I prefer to withdraw myself because I always have a feeling that no one can be trusted anymore. Many things just don't interest me anymore. Sometimes my environment appears distant and unreal and I have a feeling of “standing next to myself”, then I become totally numb. Afterwards I sometimes can't remember what has happened. I have no hope left anymore…”*

Participants were required to picture the scene and to indicate their personal assumptions concerning the described condition. Participants then responded to the Revised Illness Perception Questionnaire (IPQ-R) regarding the person described in the vignette (see below) (48).

In the focus groups, discussions were structured using an adapted version of the Short Explanatory Model Interview (SEMI; see below) (51).

#### The revised illness perception questionnaire IPQ-R

The Revised Illness Perception Questionnaire (IPQ-R) is a quantitative measure of illness perceptions (48). It has demonstrated good reliability and validity across diverse illnesses and cultures (48, 52). In the present study, we were interested in beliefs about causes of PTSD. Therefore, we used the IPQ-R subscale asking about causes (18 items) (48). Following the recommendations of Moss-Morris et al. (48), the subscale was culturally contextualized and modified to account for the characteristics of the present study. We added the item “terrible experiences” as a probable cause of PTSD, and culturally sensitive items (“God's will,” “Curse from others,” and “Evil Spirits”) in order to account for possible causal beliefs found in other studies in African samples (25, 37). The standard instruction was modified to “What are your personal assumptions and suppositions concerning the above-described discomfort of your friend?.” Participants were asked to rate their agreement with each item on a 5-point Likert scale ranging from 1 (“strongly disagree”) to 5 (“strongly agree”).

Already translated and validated versions of the English-language IPQ-R were available in German, (53) French, (54) and Arabic (55). Translation into Tigrinya was conducted using the forward and backward translation method (56).

#### Post-traumatic stress diagnostic scale PDS

Experienced traumatic events and post-traumatic symptoms were assessed using a modified version of the Post-Traumatic Stress Diagnostic Scale PDS (57). First, experienced traumatic events (23 items) were assessed using the list of the PDS extended by traumatic events from the Harvard trauma questionnaire (58) frequently experienced by asylum seekers. Afterwards, respondents rated 17 items representing the cardinal symptoms of PTSD experienced in the past 30 days on a four-point scale. These ratings summed up to a symptoms severity score ranging from 0 to 51. The cut offs are 1–10 mild, 11–20 moderate, 21–35 moderate to severe and 36 severe symptomatology (59).

#### Short explanatory model interview SEMI

Focus group discussions were moderated using key questions from the SEMI (51). The SEMI is a short interview to elicit explanatory models, exploring respondents' cultural background, nature of the presented problem, help-seeking behavior, interaction with a health care provider, and beliefs related to mental illness (51). In the present study, we were interested in causal beliefs about PTSD; therefore, only participants' responses concerning causal beliefs are analyzed in the following. The English-language SEMI (51) was translated into French and German using the method described above (56).

## Analyses

### Statistical analyses

Descriptive statistics, measures of distribution of participants' demographic characteristics, and frequency distributions of indications across the IPQ-R subscale asking about causes, were analyzed using IBM SPSS Statistics version 17.0.

To display disagreement and agreement with each of the causal IPQ-R items, values 1 (“strongly disagree”) and 2 (“disagree”) were combined, and values 4 (“agree”) and 5 (“strongly disagree”) were combined. Group differences in sociodemographic variables, traumatic events, and posttraumatic symptoms were investigated using chi-square-tests and *t*-tests (see Table 1). Statistical significance was set at *p* < 0.05 (two-tailed). Initial correlation analyses and *t*-tests revealed significant associations between religion, educational level, traumatic experiences, and symptom load on the one hand and causal attribution on the other hand in both groups. In addition to age and gender, these variables were included as covariates in the following analyses. Group differences were investigated by conducting a one-way between-groups multivariate analysis of covariance (MANCOVA). Dependent variables were items of the IPQ-R causal scale, independent variable was group—asylum seeker or no migration background. Additional analyses were conducted to check for differences between the three main countries of origin of asylum seekers: Eritrea, Somalia, and Cameroon.

Prior assumption testing was carried out to check for normality, homogeneity of variance-covariance matrices, and multicollinearity. A correlation matrix was generated to verify the absence of multicollinearity. Shapiro-Wilk tests revealed significant deviation from normal distribution in some items. However, analyses of variance were nevertheless carried out, as they have proven to be robust against deviations from the assumption of normally distributed dependent variables (60–62). Effect sizes are given as partial eta-squared as proposed by Cohen, (63) where 0.01–0.06 = small effect; 0.06–0.14 = medium effect and >0.14 = large effect.

### Interpretative phenomenological analysis (IPA)

Focus group discussions were recorded, transcribed, and analyzed using Interpretative Phenomenological Analysis (IPA) (64). The software MAXQDA version 12 was used to organize and manage data analysis.

Following the four-stage process described by Smith and Osborn, (64) the first author began with a close interpretative reading of the first transcript. Initial notes and responses to the material were registered and translated into emerging themes at one higher level of abstraction. Connections between the themes were made, which were collected in a table of superordinate themes for the first transcript (64). This procedure was repeated for each of the remaining seven transcripts. After analyses had been conducted for each of the eight transcripts, cross-case patterns were determined and recorded in a master table of themes. The identified themes were reviewed with the other researchers to ensure that the themes were well-derived from the transcripts. Moreover, the results were revised with the help of experts of the respective cultures to ensure that conclusions were culturally sensitive (64, 65).

## Results

### Sample

The sample of the questionnaire survey comprised *n* = 120 German participants without a migration background, as well as *n* = 119 asylum seekers from seven Sub-Saharan African countries (see Table 1). 71 % were male and their mean age was 28 years, which is largely in line with German asylum statistics (66). Their mean duration of residence in Germany was approximately 2 years. Participants without a migration background consisted of 35 % male participants and had a mean age of 37 years. Over half of the investigated asylum seekers were Christians and one third Muslims, while two thirds of the Germans without a migration background were Christians and almost one third stated that they were not religious. Asylum seekers indicated stronger faith and higher perceived assistance from their faith than Germans without a migration background. They had a lower education level than participants without a migration background.

The sample of the second part of the study consisted of *n* = 26 predominantly male participants (see Table 1). Their mean age was 27 years, their mean years of formal education lay at 8.5 years, and their mean duration of residence in Germany was around 1 year. Participants from Eritrea and Cameroon were all Christians, whereas participants from Somalia were all Muslims.

### Causal beliefs about PTSD—results of the questionnaire survey

Around 74% of asylum seekers and 54% of persons without a migration background had experienced at least one traumatic event (see Table 1). Asylum seekers had experienced significantly higher numbers of different traumatic events and reported significantly stronger PTSD symptoms (moderate severity) than participants without a migration background (mild severity).

The descriptive findings of the questionnaire survey showed that asylum seekers predominantly endorsed psychological causes of PTSD symptoms, such as negative thinking, stress, and the emotional state (see Table 2). Additionally, around half of them attributed PTSD symptoms to mental attitudes, family problems, terrible experiences, drug abuse, and fate. With regard to religious causes, the results demonstrated that around half of the asylum seekers attributed PTSD symptoms to God's will. In contrast, over half of them disagreed that supernatural phenomena such as curses or evil spirits could have caused the described symptoms. Furthermore, over half of the asylum seekers disagreed that organic causes, external influences, or bad luck could have caused PTSD symptoms.

**Table 2:** Causal beliefs about post-traumatic stress disorder in the group of asylum seekers.

	**Strongly disagree or disagree n (%)**	**Neither agree nor disagree n (%)**	**Strongly agree or agree n (%)**
IPQ-R CAUSE^*^		
Organic cause	67 (59.3 %)	16 (13.4%)	30 (26.5 %)
Stress or rush	25 (21.7 %)	15 (13.0%)	75 (65.2%)
Too many negative thoughts	26 (22.8 %)	7 (6.1 %)	81 (71.0%)
Hereditary	76 (66.1%)	15 (13.05)	24 (20.9%)
Emotional state	35 (30.7%)	8 (7.0 %)	71 (62.3%)
Chance or bad luck	62 (56.0%)	11 (9.5%)	40 (34.5%)
External influences	71 (62.3%)	23 (20.2%)	20 (17.5%)
Family problems	39 (34.2%)	14 (12.3%)	61 (53.5%)
Fate	45 (39.1%)	12 (10.4%)	58 (50.4%)
Mental attitude	40 (35.4%)	12 (10.6%)	61 (54.1%)
Drugs, alcohol or smoking	39 (33.9%)	14 (12.2%)	62 (53.9%)
Overwork, occupational stress	57 (49.2%)	19 (16.5%)	39 (34.0%)
God's will	44 (38.3%)	12 (10.4%)	59 (51.3%)
Curse from others	73 (62.9%)	15 (12.9%)	28 (24.1%)
Evil spirits	63 (54.3%)	18 (15.5%)	35 (30.2%)
Own behavior	49 (43.0%)	13 (11.4%)	52 (45.6%)
The personality	51 (45.1%)	15 (13.3%)	47 (41.6%)
Terrible experiences	37 (32.5%)	9 (7.9%)	68 (59.6%)

* *IPQ-R, Revised Illness Perception Questionnaire*.

Regarding the differences between the group of asylum seekers and the participants without a migration background, statistical analyses revealed significant differences for 9 out of the 18 items (see Table 3). Asylum seekers attributed symptoms more strongly to God's will, curses from others, and evil spirits, with the differences equating to large effect sizes. The attribution on God's will was stronger when the educational qualifications were higher. Muslim asylum seekers expressed a stronger belief in God's will, fate, the mental attitude, family problems, and heredity than Christian participants. Further analyses suggested that asylum seekers from Cameroon showed less belief in chance or bad luck, fate, or God's will, and persons from Eritrea showed lower beliefs in terrible experiences and occupational stress as possible causes for posttraumatic symptoms than persons from the two other groups, respectively.

**Table 3:** Inter- and Intragroup differences in causal beliefs about post-traumatic stress disorder.

	**No migration background**	**Asylum seekers**	**Intergroup differences**			**Intragroup differences**		
	**M (SD)**	**M (SD)**	**F**	**p**	**Partial η^2^**	**M (SD)**		**F**	**p**
IPQ-R CAUSE^*^									
Organic or physical cause	2.48 (1.03)	2.38 (1.34)	2.37	0.976	0.009	Eritrea	2.24 (1.27)	0.61	0.770
						Somalia	2.53 (1.29)	
						Cameroon	2.44 (1.46)	
Stress or rush	3.39 (1.06)	3.45 (1.23)	1.07	0.387	0.039	Eritrea	3.48 (0.96)	0.74	0.656
						Somalia	3.38 (1.18)	
						Cameroon	3.83 (1.15)	
Too many negative thoughts and worries^e^	3.91 (1.00)	3.63 (1.31)	1.51	0.167	0.054	Eritrea	3.28 (1.10)	0.75	0.646
						Somalia	3.75 (1.30)	
						Cameroon	3.89 (1.18)	
Heredity^a^	2.69 (1.01)	2.29 (1.27)	1.56	0.149	0.055	Eritrea	1.72 (0.84)	1.99	0.062
						Somalia	2.81 (1.23)	
						Cameroon	2.50 (1.47)	
Emotional state	3.92 (0.94)	3.43 (1.37)	2.00	0.057	0.070	Eritrea	2.88 (1.27)	1.13	0.353
						Somalia	3.63 (1.13)	
						Cameroon	3.67 (1.46)	
Chance or bad luck	2.69 (1.12)	2.67 (1.38)	1.49	0.173	0.053	Eritrea	3.12 (1.17)	2.753	0.011
						Somalia	2.72 (1.49)	
						Cameroon	1.61 (0.85)	
External influences	2.69 (1.05)	2.34 (1.31)	1.084	0.375	0.039	Eritrea	2.12 (0.97)	1.423	0.203
						Somalia	2.56 (1.59)	
						Cameroon	1.83 (0.99)	
Family problems^a^	3.74 (0.80)	3.16 (1.37)	3.43	0.002	0.115	Eritrea	2.68 (1.35)	1.09	0.383
						Somalia	3.47 (1.22)	
						Cameroon	3.67 (1.03)	
Fate^a^, ^d^	2.79 (1.17)	3.09 (1.54)	1.18	0.086	0.064	Eritrea	3.08 (1.29)	3.78	0.001
						Somalia	3.78 (1.29)	
						Cameroon	1.89 (1.23)	
Mental Attitude^a^, ^e^	3.75 (0.92)	3.18 (1.48)	2.16	0.040	0.075	Eritrea	2.64 (1.38)	1.58	0.147
						Somalia	3.81 (1.28)	
						Cameroon	3.39 (1.42)	
Drugs, alcohol or smoking	3.34 (1.12)	3.20 (1.41)	0.53	0.811	0.020	Eritrea	2.44 (1.16)	1.90	0.074
						Somalia	3.19 (1.42)	
						Cameroon	3.78 (1.17)	
Overwork, occupational stress^b^	3.65 (0.91)	2.72 (1.23)	7.96	< 0.001	0.231	Eritrea	2.12 (0.78)	2.62	0.015
						Somalia	2.97 (1.26)	
						Cameroon	3,00 (1.32)	
God's will^a^, ^c^, ^d^, ^e^	1.46 (0.76)	3.03 (1.74)	16.62	< 0.001	0.386	Eritrea	3,44 (1.45)	6.953	< 0.001
						Somalia	4.13 (1.31)	
						Cameroon	1,44 (0.78)	
Curse from others^e^	1.31 (0.56)	2.16 (1.19)	7.97	< 0.001	0.232	Eritrea	2.08 (0.95)	0.81	0.596
						Somalia	2.25 (1.19)	
						Cameroon	1.89 (1.37)	
Evil spirits^c^.^e^	1.28 (0.52)	2.53 (1.41)	13.62	< 0.001	0.340	Eritrea	2.48 (1.19)	1.509	0.171
						Somalia	2.38 (1.34)	
						Cameroon	2.61 (1.79)	
Own behavior	3.33 (0.96)	2.93 (1.36)	2.47	0.019	0.085	Eritrea	2.80 (1.19)	0.557	0.809
						Somalia	3.13 (1.34)	
						Cameroon	3.22 (1.31)	
Personality^b^	3.58 (0.85)	2.84 (1.24)	4.15	< 0.001	0.136	Eritrea	2.68 (1.28)	1.097	0.377
						Somalia	3.06 (1.27)	
						Cameroon	3.00 (1.19)	
Terrible experiences	4.44 (0.58)	3.40 (1.43)	7.30	< 0.001	0.216	Eritrea	2.80 (1.26)	2.608	0.015
						Somalia	3.72 (1.20)	
						Cameroon	4.22 (1.26)	

* *IPQ-R = Revised Illness Perception Questionnaire*.

a *significant influence of religion in the group of asylum seekers*.

b *significant influence of gender in the group of asylum seekers*.

c *significant influence of gender in the group of Germans without a migration background*.

d *significant influence of education in the group of asylum seekers*.

e *significant influence of education in the group of Germans without a migration background*.

Participants without a migration background attributed symptoms more strongly to psycho-social causes, such as the mental attitude, one's own personality and behavior, family problems, and occupational stress (medium effect sizes). Furthermore, participants without a migration background attributed symptoms more strongly to terrible experiences (large effect size). Female participants without a migration background attributed symptoms more strongly to God's will and evil spirits when their educational qualifications were higher.

### Causal beliefs about PTSD—themes emerging in the focus group discussions

Participants of all focus group discussions identified the described PTSD symptoms either in themselves or in somebody they knew. Regarding causal beliefs about PTSD, the majority of participants did not indicate a single firm assumption. Various causal beliefs were stated, which also formed part of participants' etiological belief systems. These causal beliefs can be grouped into six superordinate themes: (a) traumatic life experiences, (b) psychological causes, (c) social causes, (d) post-migration stressors, (e) religious causes, and (f) supernatural causes.

#### Traumatic life experiences

Respondents' own life experiences before and during their migratory trajectories were the most prevalent causes attributed to the vignette (*n* = 22).

“In your own home country you are in prison. I was in military service for 18 years. (…) Then came the time of war and riots where I suffered my first shock, the ruthlessness of war.” (Man, 35 years, Eritrea).

Participants described their flight as a time of extreme adversity and the trajectories as grueling and unmerciful; a matter of life and death. Across all focus groups, participants reported episodes in which they were threatened with weapons, persecuted, or abducted. They especially emphasized the violence against women and described systematic separation, widespread rape, and sexual violence against women (*n* = 10). Participants identified three major stages that they considered to be particularly frightening during their flight: the crossing of the Sahara; their stay in Libya or Morocco; and the crossing of the Mediterranean Sea. The following quotes exemplify participants' experiences:

“I saw some women that experienced violence. I was beaten. Some others were probably raped.” (Woman, 34 years, Eritrea)

“And what has happened to me in Libya. These fears, when people with knives were coming behind us.” (Man, 31 years, Eritrea)

“My boat capsized two times. People died. I saw somebody with whom I was talking, dying.” (Man, 23 years, Cameroon)

“These experiences force you to become unscrupulous and you will lose your belief in humanity. I saw what the Bedouins did to them. They took organs from people who were fully conscious.” (Woman, 35 years, Eritrea)

#### Psychological causes

Participants across the focus groups indicated mental or psychological causes of the described condition. Some participants identified the condition as trauma (*n* = 3) or depression (*n* = 6). Participants from Cameroon and Somalia described the person in the vignette as crazy or mad; while participants from Cameroon used the French expression *Folie*, participants from Somalia used the expression *Wali* (being mad).

Stress (*n* = 26) was emphasized as one major cause of the described symptoms. While asylum seekers from Cameroon used the French word *Stress*, participants from Somalia used the expression *Isku buukh* (stress):

“*Isku buukh*. Makes you sick. Stress makes you a mental problem. Like this problem. Can make you totally mad.” (Man, 24 years, Somalia)

Participants from Eritrea agreed on the concept of *Chinket* (stress), which is used synonymously to describe stress or depression. They identified *Zekta* (feeling of pressure) as a cause of *Chinket* (stress):

“*Zekta*, this feeling of suppression, causes *Chinket*.” (Woman, 35 years, Eritrea)

Participants (*n* = 8) across focus groups described *thinking too much* as one cause of the symptoms. Participants from Somalia used the expression *Murug* (thinking too much).

#### Social causes

Participants strongly perceived themselves to be members of social and cultural frameworks and referred to their social positions within the collective. These social systems included their families, their village communities, and their tribes or clans. In their new society of resettlement, they felt ripped out of their social communities and excluded. Participants greatly missed their families who had remained at home or had resettled in other countries. They expressed their feelings of loneliness and distance. Participants of all focus groups identified these feelings of isolation as one cause of PTSD:

“And I see this as a cause for this stress, the missing community and the life in a completely new environment.” (Man, 35 years, Eritrea).

Furthermore, participants (*n* = 8) perceived a high degree of incongruity between their responsibility to provide for their families at home, their moral values, and the complex challenges they are facing in their society of resettlement. Therefore, they attributed the symptoms to their inability to adhere to their intergenerational obligations:

“It's a little bit like a debt. Your parents lend to you today, you will return later. (…) Because you have left a family in Africa and you know the conditions of life. (…) You are not calm. You don't have the power to help them.” (Man, 28 years, Cameroon)

“I don't have money. Where can I get money? My mother calls me. (…) Can you send us money? (…) How do you feel when your mother tells you she doesn't have anything to eat?” (Man, 30 years, Somalia)

#### Post-migration stressors

Participants across the groups emphasized their new life circumstances in Europe as one major cause of the PTSD symptoms. Confronted with the reality of life in Europe, they felt disappointed and disillusioned. Moreover, they described their state of impotence and paralysis due to their strong concerns about a potential rejection of their application for asylum. Without language competences and work opportunities, they felt helpless, dependent, and other-directed. They were particularly preoccupied by financial problems (*n* = 10):

“With the asylum procedure, you see, he can't eat. They send maybe a letter, where they say, ok Sir; you have to leave the country. (…) He is scared that he might sleep and the police will take him. So he sleeps in the woods. (…) This is why we find people who manifest this kind of behavior.” (Man, 28 years, Cameroon)

“I know most of us in Africa consider Germany as a heaven. But it's also a hell for us.” (Man, 30 years, Somalia)

#### Religious causes

Participants felt strongly embedded in their religious convictions and communities. The importance of religion for mental health and illness was frequently stressed during the discussions. Participants strongly highlighted religious causes of PTSD symptoms (*n* = 26), including lack of faith and adherence to religious commandments as well as sinful behavior. In this vein, participants from Eritrea (*n* = 3) reported the concept of *Mijgab* (lack of faith and religious gratitude) as a cause. Participants across all focus groups discussed the attribution of the described symptoms to God's will or *Sheitan* (the devil):

“Yes clearly. For me, this stress is related to religion (…) and to the devil. *Sheitan*.” (Man, 35 years, Eritrea)

“Furthermore, I believe very strongly that it's somehow God's will (…).” (Man, 31 years, Eritrea)

#### Supernatural causes

Participants across the focus groups described supernatural phenomena as causes of PTSD symptoms. While there were differences in belief systems among participants of the different countries of origin, remarkable similarities in concepts within the supernatural realm were observed. Witchcraft, being cursed, and possession by evil spirits were reported as causes of PTSD symptoms. However, these were subject to controversial debate in the religious context and often perceived as interrelated with the devil and as opposed to a life within religious precepts.

##### Spirit possession

Respondents from all three countries of origin described the possession by evil spirits as possible causes of PTSD symptoms. These included a failure to honor ancestral spirits, spirits within an Islamic context, or bad spirits from nature.

Several participants from Cameroon (*n* = 4) believed the person described in the vignette to be under spiritual attack, and the interrelation of evil spirits (*des mauvais esprits*) and witchcraft (*la sorcellerie*) was discussed:

“It's an evil spirit that is disturbing him.” (Man, 23 years, Cameroon)

“All of this is dark, because witchcraft and evil spirits, these two are the same. So, when you are talking of witchcraft, you are disturbed by people. (…) Maybe they will attack you spiritually.” (Man, 20 years, Cameroon)

Some participants from Somalia (*n* = 5) attributed PTSD symptoms to a possession by *Djinn* or *Gini* (spirits). They explained that *Gini* could be either good or bad and referred to the Koran. They were believed to inhabit an invisible parallel world, from where they are able to observe the human world:

“*Sheitan* is *Gini*. (…) A beautiful girl like this becomes sick. That is *Gini*.” (Man, 26 years, Somalia)

“There are verses in the Koran whereby you can read, someone who has *Djinn*, the person will weep, the person will cry (…). Sometimes *Djinn* lives in different parts of the body. They can live in the pinky finger. They can live in the head. They can live everywhere.” (Man, 30 years, Somalia)

According to some participants from Eritrea (*n* = 3), different types of evil spirits were perceived to be the cause of illness and suffering within the concept of *Idnaisep* (the hand of another):

“*Idnaisep*, he is possessed by the illness of somebody else. Something like an evil spirit entered him.” (Man, 25 years, Eritrea)

Referring to their migratory trajectories, some participants from Eritrea (*n* = 4) attributed PTSD symptoms to *Megagna* (type of evil spirit in the desert and the Mediterranean Sea), who causes *Likift* (the corresponding illness):

“This is one of the bad spirits, we call it *Megagna*. (…) If he gets you, this bad spirit and you will get this illness. (…) We all crossed the middle sea. In the sea sometimes, (…) we don't know how long a day we traveled. So sometimes we get this *Megagna* and a lot of people they throw themselves in the sea.” (Man, 29 years, Eritrea)

##### Curses and bedevilment

Curses and bedevilment were discussed as other possible causes of the described PTSD symptoms. The belief in curses was often strongly associated with the belief in evil spirits, and the constructs tended to be merged into one another rather than being clearly separable. Cursing was described as a practice originating from other individuals or through the mediation of traditional healers and sorcerers. Across all focus groups, participants reported that cursing was often performed within a family conflict or motivated by jealousy toward successful family members:

“Problems between the brothers, problems between the parents and their children, and then you see somebody who has these personal problems. (…) He gets sick. At home you would maybe say, it's his brother with whom he has these problems. He is practicing witchcraft or evil spirits on him.” (Man, 25 years, Cameroon)

Some participants from Eritrea (*n* = 4) attributed symptoms to a curse referred to as *Buda* (evil eye). Unrequited love, being unfaithful within marriage, and jealousy were discussed as motives for such an attack. The transmission of *Buda* occurs through the eyes:

“There are a lot of *Budas*. (…) When it attacks somebody that spirit for example. That spirit attacked me. I start crying and shouting.” (Man, 35 years, Eritrea)

Participants across the focus groups described that the practice of cursing was often associated with disobedience, their incapacity to support their families remaining at home, and the disregard of the requests of their social origin group. Within this form of cursing, transmission was believed to happen from one person to another through the mind or the heart:

“And they will ask you to come back to your village and you refuse. All these curses (*Malédictions*) will follow me because I disobeyed.” (Man, 26 years, Cameroon)

Participants from Somalia applied the term *Habaar* (curse), referring to a form of cursing that can be applied after the refusal of support. This form of curse was explained to originate from disrespectfully treated or ignored individuals, with the desire to cause misfortune:

“Because now you came from a family that in terms of status (…) is low class. But when they see that we live in Germany. (…) My son doesn't send me anything. The parents send some sort of *Habaar* to you. (…) is a term that is used when you ignore things or you ignore people. Then, systematically it comes from the heart of this people. You know they say that, may God not bless your health. Then you become this.” (Man, 30 years, Somalia)

## Discussion

The present study aimed to provide insights into beliefs about causes of PTSD held by Sub-Saharan African asylum seekers residing in Germany. Their causal beliefs were compared to those held by a German population without a migration background, and the role of supernatural and religious causation in explaining PTSD symptoms was examined.

In the quantitative part of the study, descriptive analyses demonstrated that Sub-Saharan African asylum seekers attributed PTSD symptoms mainly to psychological causes. Furthermore, they attributed symptoms to religious causes, family problems, terrible experiences, drug abuse, and fate. The majority of the participants rather disagreed with the concept of supernatural phenomena, such as curses and evils spirits, as causes of PTSD symptoms.

Regarding the sample comparisons, analyses revealed consistent differences between Sub-Saharan African asylum seekers and participants without a migration background. Asylum seekers attributed symptoms more strongly to religious and supernatural causes compared to participants without a migration background. However, analyses within the group of asylum seekers indicated that participants from Cameroon rarely attributed symptoms of PTSD to God's will. In this regard, their belief was more similar to German participants than to other participants from Sub-Saharan Africa. Nevertheless, our results are largely in line with previous quantitative research, which reported corresponding differences between Western samples and samples from West Africa, (27) Cameroon, (33) and Nigeria, (26) with African respondents more often favoring supernatural causes of mental disorders.

With regard to the qualitative part of the study, we identified six superordinate themes which we consider to be crucial in understanding causal beliefs about PTSD among Sub-Saharan African asylum seekers. Participants of the focus group discussions predominantly indicated their own traumatic life experiences, psychological and social causes, as well as post-migration stressors, as causes of PTSD symptoms. They also discussed religious and supernatural causes, although these were controversially debated.

The findings of the two parts of the study are consistent in demonstrating that Sub-Saharan African asylum seekers attribute the symptoms of PTSD to internal psychological factors, such as experiencing stress, being in a negative emotional state, and thinking too much, rather than to internal physical mechanisms related to organic or genetic factors. As expected, our findings underline the importance of traumatic experiences before and during migratory pathways in explaining PTSD symptoms among asylum seekers. Accordingly, the symptoms were explained by the accumulation of hardships and experiences of poverty and war (67). Moreover, stressful life circumstances in asylum seekers' new society of resettlement need to be emphasized. These were often linked to social factors such as family problems and the asylum seekers' inability to adhere to their intergenerational obligations toward their families left at home. The incapacity to support their families financially was perceived to be especially burdensome. The arising feelings of guilt were often paired with their social isolation and feelings of loneliness.

Nevertheless, and in line with previous research [e.g., 31], religion did play a significant role in explaining Sub-Saharan African asylum seekers' beliefs about the causes of PTSD symptoms. This should be understood as an external symptom attribution and therefore as an attempt to find a culturally shared and familiar explanation, which is opposed to the impression of being helplessly exposed to the symptoms. Finding a culturally shared and accepted meaning for aversive experiences should be considered as a protective factor. Our findings suggest that when diagnosing and treating immigrants from Sub-Saharan African cultures, it might be crucial for clinicians to explore a religious dimension within patients' belief systems in order to enhance therapeutic alliance and improve treatment outcome (35, 68). Moreover, our findings may have implications with respect to help-seeking behavior and treatment preferences of this particular group. In this line, previous research demonstrated that refugees and asylum seekers in Western countries showed clear preferences for religious treatment practices in the context of mental illness (34, 42, 69).

With regard to supernatural causation, the results of the two parts of the study were rather divergent. While many respondents of the questionnaire survey disagreed with supernatural causes of PTSD symptoms, subsequent differences to the German comparison sample were found and participants of the qualitative part discussed cursing and evil spirits as culturally accepted causal beliefs. Our findings therefore suggest that while supernatural causation must be considered as a culturally acceptable explanatory approach among asylum seekers from Sub-Saharan Africa, its role in the context of explaining PTSD symptoms should not be overstated. Nevertheless, our findings can help Western professionals in some cases to better understand this group of patients and differentiate culturally shaped attributions of PTSD symptoms from psychotic symptoms, and ultimately reduce false diagnoses (41). The present findings seem to differ from previous research that emphasized the role of supernatural causal beliefs in explaining mental disorders (31). We presume that one reason for this occurrence might be the encounter of Western culture. The literature has described migration, globalization, and urbanization as diminishing supernatural explanations for mental illness (32, 34, 70). As previous research often focused on in-depth information collected mainly by qualitative methods, (21, 36, 37) we assume that our findings might differ from previous work because of the inclusion of a quantitative research approach.

By combining a qualitative with a quantitative research approach, the present study complements and extends previous quantitative and qualitative work on illness beliefs of refugees and asylum seekers from different regions of Sub-Saharan Africa (21, 35–38). By drawing data from different sources, the perspective was broadened and a deeper insight into causal beliefs about PTSD held by asylum seekers from Sub-Saharan Africa was possible. The review of the results of the survey study within focus group discussions helped in contextualizing and interpreting the quantitative results and ensured a culturally sensitive interpretation of the questionnaire items. For instance, this was particularly apparent with regard to the item family problems. While Western researchers and clinicians would assign an interpretation that would rest on an interpersonal level, asylum seekers from Sub-Saharan Africa clearly added a supernatural meaning. According to their reasoning, family problems might signify that supernatural practices had been performed by parents, family members, or through the mediation of bad traditional healers and sorcerers. Jealousy, disobedience or disrespect of parents or elders were stated as the main causes of supernatural practices within a family context. Therefore, talking about supernatural practices can be interpreted as a form of expressing and explaining negatively appraised feelings such as aggression, guilt, and jealousy, as well as conflicts within one's own family.

In general, our study demonstrated consistent differences between asylum seekers and Germans without a migration background. However, at the same time, remarkable similarity concerning attribution on psychological causes emerged. The psychological causal beliefs found in the present study corresponded remarkably well to the Western perspective on causes of PTSD symptoms. Our results therefore reveal that the current Western understanding of PTDS is as relevant to migrants as to non-migrants in some respects. While awareness of culture-specific causation is important, at the same time, clinicians need to beware of cultural stereotyping (40). It is necessary to acknowledge the diversity of belief systems and attitudes within cultures and to attempt to understand each individual context. Cultural sensitivity in psychotherapy therefore means facing each individual's system of beliefs in an empathic and non-judgmental way (40).

## Limitations

A first limitation of the present study lies in the diverse sample. We investigated a diverse group of asylum seekers with regard to countries of origin and cultural groups of Sub-Saharan Africa and different religions. While we accounted for differences with regard to the main countries of origin and several sociodemographic factors, illness perceptions may still vary due to differences in social positions and languages (71). Therefore, conclusions about the impact of culture on participants' causal beliefs remain limited.

Second, characteristics of the two investigated groups in the quantitative part differed in terms of age, gender, education, religion, traumatic experiences, and PTSD symptoms. Even though these variables were included in the analytical model to control for their influence, conclusions about the influence of culture should be drawn with caution.

Third, we used a general population approach by applying a case vignette design. The investigated groups differed in terms of experienced traumatic events and post-traumatic symptoms, and we did not control for prior treatment experience. Furthermore, we used different traumatic events in the vignettes for the two groups, in order to account for the different living situations and the different probabilities for experiencing the respective traumatic event in each group. However, while all asylum seekers fled from their home countries and thus have experienced a flight, not all Germans without a migration background have experienced or witnessed a physical attack: it was among the six most frequently indicated traumatic events (17.5%, *n* = 21) in their group. This difference in experiencing the described traumatic event and posttraumatic symptoms might have led to different answers regarding causes of PTSD. For future studies, it might be interesting to include a clinical sample and compare responses to those of lay people.

Forth, the recruitment of participants may have encompassed a selection bias, as study participation required competences in specific languages, and excluded persons who only spoke Somali, for example.

Fifth, focus group discussions with predominantly male participants were conducted by a white, female clinical psychologist, which may have resulted in a response bias. The fact that a non-African member of the majority society is showing an interest in the perceptions of asylum seekers from Sub-Saharan Africa might be classified as a social act, which might have induced social desirability in the responses (31). Although we presume that participants spoke openly without reservations, some may have been reticent to share certain opinions as a result of perceived differences in gender and cultural background or social desirability.

Sixth, as our participants were predominantly male, we present a particularly male perspective of causal beliefs about PTSD symptoms. Thus, we cannot rule out an impact of gender on our results. It would be interesting for future research to explore gender differences in causal beliefs about PTSD.

## Conclusion

Our findings suggest that the current Western understanding of PTSD is as relevant to migrants as to non-migrants in terms of psychological causation, but might differ regarding the religious and supernatural realm. While awareness of culture-specific causal beliefs of asylum seekers from Sub-Saharan Africa regarding PTSD is important, our findings do underline at the same time that cultural differences should not be overstated. Exploring patients' individual perspectives can help clinicians to provide better patient-centered care and to tailor interventions according to patients' culturally shaped belief systems (72). For instance, if a clinician shows a willingness to incorporate a religious dimension into psychotherapy, this might enhance the patient's therapy motivation and engagement in treatment. Moreover, we argue that future studies in other immigrant populations should employ the triangulation of quantitative and qualitative methods in order to ensure a culturally sensitive research practice.

## Ethics statement

Approval for the study was obtained from the local Ethics Committee at the Department of Psychology at Philipps-University Marburg, Germany. All participants provided informed consent to take part in the study.

## Author contributions

FG analyzed and interpreted the data, and was a major contributor in writing the manuscript. MRM, UN, and SS contributed to the interpretation of data and critically revised earlier versions of the manuscript. RM was the senior principal investigator of the study, gave feedback to the analyses and the interpretation of the data, and was a major contributor in writing the manuscript. All authors read and approved the final manuscript.

### Conflict of interest statement

The authors declare that the research was conducted in the absence of any commercial or financial relationships that could be construed as a potential conflict of interest.
